# Exploring the crosstalk between endothelial cells, immune cells, and immune checkpoints in the tumor microenvironment: new insights and therapeutic implications

**DOI:** 10.1038/s41419-023-06119-x

**Published:** 2023-09-04

**Authors:** Jianwen Fang, Yue Lu, Jingyan Zheng, Xiaocong Jiang, Haixing Shen, Xi Shang, Yuexin Lu, Peifen Fu

**Affiliations:** 1 Department of Breast Surgery, The First Affiliated Hospital, Zhejiang University School of Medicine, 310003 Hangzhou, China; 2 Department of Breast and Thyroid Surgery, First Affiliated Hospital of Huzhou University, 313000 Huzhou, China; 3 Department of Breast and Thyroid Surgery, Lishui People’s Hospital, The Six Affiliated Hospital of Wenzhou Medical University, 323000 Lishui, China; 4 Department of Breast and Thyroid Surgery, Cixi People’s Hospital, 315300 Cixi, China; 5 Department of Breast and Thyroid Surgery, Taizhou Hospital, Zhejiang University, 318000 Taizhou, China

**Keywords:** Cancer microenvironment, Cell signalling

## Abstract

The tumor microenvironment (TME) is a highly intricate milieu, comprising a multitude of components, including immune cells and stromal cells, that exert a profound influence on tumor initiation and progression. Within the TME, angiogenesis is predominantly orchestrated by endothelial cells (ECs), which foster the proliferation and metastasis of malignant cells. The interplay between tumor and immune cells with ECs is complex and can either bolster or hinder the immune system. Thus, a comprehensive understanding of the intricate crosstalk between ECs and immune cells is essential to advance the development of immunotherapeutic interventions. Despite recent progress, the underlying molecular mechanisms that govern the interplay between ECs and immune cells remain elusive. Nevertheless, the immunomodulatory function of ECs has emerged as a pivotal determinant of the immune response. In light of this, the study of the relationship between ECs and immune checkpoints has garnered considerable attention in the field of immunotherapy. By targeting specific molecular pathways and signaling molecules associated with ECs in the TME, novel immunotherapeutic strategies may be devised to enhance the efficacy of current treatments. In this vein, we sought to elucidate the relationship between ECs, immune cells, and immune checkpoints in the TME, with the ultimate goal of identifying novel therapeutic targets and charting new avenues for immunotherapy.

## Facts

1. The interaction between endothelial cells and immune cells in the tumor microenvironment can influence immune cell infiltration and function.
2. Endothelial cells can express immune checkpoint molecules, such as PD-L1, which can modulate immune cell activity.
3. Combination therapies targeting both the tumor vasculature and the immune system hold great potential for enhancing treatment response and overcoming resistance in cancer therapy.

## Open questions

1. What are the molecular mechanisms underlying the crosstalk between endothelial cells and immune cells, and how can we target these interactions to enhance the efficacy of immunotherapy?
2. What is the role of endothelial cell-expressed immune checkpoint molecules in regulating immune cell function?
3. Can we develop strategies to overcome the immunosuppressive effects of the tumor microenvironment and enhance the activity of immune cells in the presence of antiangiogenic therapy?

## Introduction

During phases of rapid growth, tumors exhibit a high degree of vascularization, while dormant tumors do not [1]. This highlights the critical role of angiogenesis in tumor progression, as it enables the delivery of oxygen, nutrients, and growth factors to distant organs, as well as the spread of cancerous cells [2]. The process of angiogenesis is regulated by a complex interplay of signaling molecules and pathways within the tumor microenvironment (TME) [3]. At the core of this process lies the proliferation, migration, and morphogenesis of endothelial cells (ECs) [4]. Numerous angiogenic regulators associated with the process of angiogenesis involve surface receptors on ECs and their corresponding ligands. Among these crucial ligands and receptors are the VEGF/VEGFRs and the angiopoietin and Tie2 pathways [5]. Within the human body, ECs form the inner lining of large blood and lymph vessels, as well as the microvasculature [6]. Despite their relatively small proportion of tissue cells, ECs are essential for maintaining tissue homeostasis, regulating blood vessel dilation, blood coagulation, and promoting angiogenesis [7]. Moreover, ECs play a critical role in the circulatory system by providing paracrine support to surrounding nonvascular cells, regulating permeability and inflammation, and maintaining the balance between coagulation and fibrinolysis [8, 9].

Impaired endothelial barrier function has been implicated in the morbidity and mortality associated with a range of pathological conditions, such as sepsis, pulmonary edema, and reperfusion injury [10]. In addition to regulating blood fluidity and platelet adhesion, ECs also play a key role in controlling leukocyte activation, adhesion, and transmigration [11]. They are central to regulating immunity and inflammation, as well as balancing coagulation and fibrinolysis [12]. The activation of leukocytes motility is facilitated by the binding of adhesion molecules, such as selectins [13]. Notably, ECs express E-selectin and P-selectin, which serve as pivotal molecules in mediating this process [14]. Evidence suggests that ECs are plastic and can undergo a transition to mesenchymal cells through a process known as endothelial-to-mesenchymal transition (EndMT) [15]. In the context of cancer, EndMT is a critical adaptation of the TME that promotes cancer proliferation, spreading, and resistance to chemotherapy [16]. ECs undergo EndMT to shed endothelial characteristics and acquire mesenchymal markers, accompanied by greater transcription factor expression, defaulting to a mesenchymal cell function and exhibiting some mesenchymal cell characteristics, as well as the loss of their capability to form capillaries and cell-cell junctions, the increase in cellular migration properties, and the release of extracellular matrix proteins as a result [17]. The concerted action of TGF-β and TNF-α drives the induction of EndMT, wherein ECs that have undergone this process autonomously secrete TGF-β2 and Activin [18]. These secreted cytokines not only reinforce the mesenchymal phenotypes of ECs, but also induce the profound epithelial-to-mesenchymal transition (EMT) in epithelial cancer cells [18, 19].

Immune cells present within the TME play a crucial role in recognizing and eliminating cancer cells during the early stages of tumor development [20]. However, their function eventually becomes restricted through various mechanisms. In a recent study, an intriguing tumor T-cell metabolic circuit has been identified, wherein tumor-derived lactate disrupts the pyruvate metabolism of CD8^+^ T cells, consequently impeding the cytotoxicity of these cells [21]. There is still much to be unraveled regarding intricate mechanisms underlying tumor immunosuppression such as this. ECs and immune cells engage in complex communication via immune targets, adhesion, and signaling pathways [22]. The precise impact of ECs on immune cell function within the TME remains incompletely understood, and the underlying mechanisms of their interaction represent a potential target for cancer therapies. In this study, we endeavored to delve into, investigate, and establish the intricate connection between ECs and immune cells, thereby elucidating promising avenues for therapeutic interventions.

Immune checkpoint molecules, including inhibitory and stimulatory checkpoint molecules, are expressed on both adaptive and innate immune cells [23]. The emergence of a new generation of immune checkpoint inhibitors (ICIs) has revolutionized tumor treatment, offering patients with metastatic disease the opportunity to live longer and providing new therapeutic indications in earlier stages of cancer [24]. The link between immune checkpoints and ECs is an important consideration in the TME and may have implications for cancer treatment.

The aim of this paper is to investigate the critical role of ECs in the TME, including their interaction with immune cells, their relationship with immune checkpoints, and their impact on immunotherapy. Furthermore, we will explore the potential to enhance the efficacy of immunotherapy by targeting ECs.

## Exploring the role of ECs in the TME

### TME: a dynamic ecosystem driving oncogenesis and metastasis

TME constitutes a complex and intricate multicellular milieu that nurtures cancer development [25]. The cellular components residing within the TME are postulated to exert a profound influence on fundamental cancer hallmarks, including proliferation, angiogenesis, invasion, metastasis, and most notably, resistance to chemotherapy [26]. These multifarious functions are orchestrated by a diverse array of cell types, including tumor cells, immune cells, fibroblasts, and endothelial cells, along with secreted factors that collectively constitute the TME. As such, they present themselves as potential targets for anticancer therapies [27]. The cancer-associated fibroblasts (CAFs) have been found to secrete hepatocyte growth factor (HGF), thereby instigating resistance to pharmacological interventions targeting tyrosine kinases and the epidermal growth factor receptor (EGFR) [28]. ECs also participate in promoting tumor resistance through various mechanisms. Studies revealed that chemotherapeutic drug exposure triggers ECs to secrete TNF-α and promotes CXCL1/2 expression in cancer cells, contributing to amplification of CXCL1/2-S100A8/9 loop and inducing the acquisition of therapeutic resistance [29]. Recognizing the dynamic changes occurring in the TME during tumor progression could lead to treatment strategies that can address each stage of tumor evolution [30]. In recent years, the strides achieved in molecular biology research have given rise to the advent of molecular targeted therapies and immunotherapies, exemplified by the inhibition of PD-1/PD-L1 signaling [31]. Such interventions aimed at the TME include blocking extracellular ligand-receptor interactions and downstream signaling pathways, leading to the discovery of novel targets that enhance the efficacy of numerous cancer therapies [32]. Notably, immunotherapies that augment the host’s anti-tumor immune response by targeting TME have emerged as a promising approach [33]. Among the factors that promote angiogenesis, TME plays a pivotal role [34]. Proliferation and motility of ECs are crucial for angiogenesis, which are associated with tumor progression and metastasis [35]. Targeting the metabolism of ECs represents a promising strategy for treating diseases, including cancer [36]. Bevacizumab (Avastin), an anti-VEGF monoclonal antibody, inhibits the VEGF signaling pathway, which is pivotal in promoting tumor growth and metastasis through angiogenesis [37]. It has been widely used in combination with chemotherapy as the first antiangiogenic drug for multiple malignancies.

### Hypoxia as a key regulator of tumor progression in the TME

Hypoxia, one of the hallmarks of TME, stimulates the production of pro-angiogenic factors by tumor cells, particularly vascular endothelial growth factor (VEGF), resulting in the activation of the “angiogenic switch” [38]. This triggers the proliferation and migration of ECs, leading to the formation of new blood vessels [39]. Hypoxic microenvironments are present in most tumors and promote tumor progression and therapeutic resistance by enhancing abnormal angiogenesis, desmoplasia, and inflammation [40]. Under hypoxic conditions, hypoxia-inducible factors (HIF), which regulate genes such as VEGF, transforming growth factor (TGF-β), platelet-derived growth factor B (PDGF-B), plasminogen activator-1 (PAI-1), and erythropoietin (EPO), escape degradation and bind to hypoxia response elements (HREs) [41]. This facilitates the expression of genes required for cellular response in overcoming hypoxia, including angiogenesis. To persist in the hypoxic microenvironment, cancer cells induce multiple biological pathways that are mediated by HIF [42]. These pathways encompass metabolic reprogramming, cellular survival, and migration. Notably, the activation of HIF-dependent genes, which encode crucial glycolytic enzymes (such as hexokinase 1 and 3, aldolase A and C, and glyceraldehyde 3-phosphate dehydrogenase), as well as the monocarboxylate transporters responsible for glucose (glucose transporters 1 and 3) and lactate, are prominent examples of the molecular players implicated in these intricate mechanisms [42]. By orchestrating the migration of ECs to hypoxic areas, HIF facilitates the formation of new vascular networks to overcome oxygen deficiency [43]. In this intricate process, the activation of HIF-1α initiates the transcriptional upregulation of VEGF in tumor cell, thereby orchestrating the recruitment of ECs to hypoxic regions and stimulating ECs proliferation [44].

### VEGF signaling in TME modulation

The VEGF/VEGFR axis is widely recognized as a crucial driver of tumor vascularization, as evidenced by various studies [45]. Oncogene activation and tumor suppressor gene mutations also contribute to VEGF upregulation in cancer cells [46]. Through paracrine signaling, VEGF activates ECs and stimulates their migration and proliferation, ultimately resulting in angiogenesis [47]. The expression of VEGF is commonly observed in many malignant tumors and is generally considered to be a major driver of tumor angiogenesis [48]. It has been proposed that VEGF signaling modifies the TME by indirectly enhancing tumor cell migration, intravasation, and survival, as well as promoting tumor metastasis and vascular permeability [49]. Notably, VEGF exerts various effects on immune cells, such as inhibiting T-cell function, increasing regulatory T cells (Tregs), and inhibiting dendritic cell (DC) differentiation and activation [50]. Since the discovery that bevacizumab is effective in treating metastatic colorectal cancer [51], VEGFR-targeting molecules have been approved for the treatment of various tumors [52]. However, it is important to note that resistance to antiangiogenic drugs often arises during clinical treatment, ultimately resulting in unfavorable treatment outcomes and failure [53]. Despite the limited understanding of potential predictive biomarkers and mechanisms of response and resistance, the clinical combination of antiangiogenic therapy with ICIs has yielded significant advancements in the treatment of metastatic cancer [54].

### Angiogenic factors beyond VEGF: driving improper vascular network formation in tumors

In addition to VEGF/VEGFR signaling, other growth factors can also contribute to the formation of aberrant tumor vasculature. For instance, members of the angiopoietin (Ang), PDGF-B, and TGF-β families have been implicated in this process [55]. While Ang-1 mediates migration and adhesion of ECs, Ang-2 inhibits the communication between the endothelium and perivascular cells thereby facilitating vascular regression [56]. However, when combined with VEGF, Ang-2 can promote angiogenesis [56]. The Tie2 receptor tyrosine kinase, which is expressed in ECs, tumor-associated macrophages (TAMs), and tumor cells, binds to the ligands Ang-1 and Ang-2 [57]. Hypoxia increases the expression of Tie2 in monocytes, which, in combination with Ang-2, suppresses their anti-tumor functions [58]. Similarly, PDGF-B promotes the growth, invasion, and angiogenesis of tumor cells, leading to tumor metastasis in multiple cancer types [59]. TGF-β, on the other hand, plays diverse roles in the TME, including stimulating the differentiation of ECs and myofibroblasts, recruiting immune cells, and inhibiting anti-tumor immune responses [60].

### ECs and tumor progression: a complex interplay

**Fig. 1:**
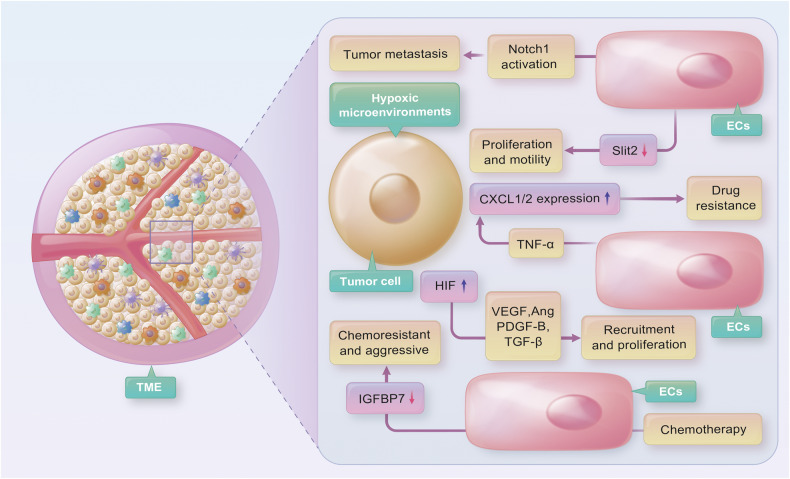
Tumor cells secrete several angiogenic factors (e.g., VEGF, ANG, PDGF-B, TGF-β) that promote the proliferation of endothelial cells (ECs). A hypoxic environment potentiates the ability of tumor cells to stimulate angiogenesis via hypoxia-inducible factors (HIFs). Tumor cells activate Notch1 signaling in ECs, facilitating tumor cell metastasis. Additionally, ECs downregulate Slit2, promoting tumor proliferation and motility. Exposure to chemotherapeutic drugs induces ECs to secrete TNF-α and enhances CXCL1/2 expression in cancer cells, leading to the development of therapeutic resistance. Furthermore, chemotherapy suppresses the expression of IGFBP7 in ECs, resulting in the emergence of aggressive and chemoresistant tumor cells.

During the formation of blood vessels in the TME, ECs are exposed to an unique milieu of extracellular signals, including hypoxia, fluctuating blood flow, low pH, and growth factors and cytokines released by tumor cells [61]. In turn, ECs can release factors that affect tumor cell adhesion, promoting tumor growth and invasion [61, 62]. The interaction between tumor cells and ECs is closely associated with tumor growth and metastasis [63]. For instance, when ECs undergo chemotherapy, insulin-like growth factor binding protein-7 (IGFBP7/angiomodulin) expression is suppressed, resulting in the emergence of chemoresistant and aggressive tumor cells, which potentially contribute to tumor progression and metastasis [64]. Tumor cells can also activate Notch1 signaling activity in ECs, leading to persistent Notch1 activation that facilitates tumor cell transmigration, intravasation, and metastasis [65]. Furthermore, ECs downregulate Slit2, a tumor-suppressive angiocrine factor inhibited by EphA2, thereby facilitating tumor proliferation and motility [66] (Fig. 1). ECs are not functionally fixed and can differentiate into other cell types, such as fibroblasts, chondrocytes, and osteoblasts [67]. Evidence suggests that ECs can differentiate into osteoblasts in the bone microenvironment during prostate cancer bone metastasis [68].

### Endothelial progenitor cells: implications for tumor angiogenesis and beyond

After the release of tumor-secreted cytokines, endothelial progenitor cells (EPCs) migrate from the bone marrow to the bloodstream, infiltrate into the tumor mass, differentiate into ECs, and produce angiogenic factors that promote tumor vascularization [69]. The amount of circulating EPCs in the body is strongly correlated with cancer progression [70]. In addition, EPCs are recruited into the tumor mass and contribute to the “angiogenic switch”, either directly by integrating into cancer vessels or indirectly by secreting pro-angiogenic cytokines paracrinely [57]. Apart from their role in neovascularization, EPCs have promising therapeutic and prognostic potential for malignant tumors [71]. Previous research has suggested that monocytes may also serve as EPCs, expressing EC markers and integrating into blood vessels to promote tumor progression [72]. Tumor-secreted cytokines such as monocyte chemoattractant protein 1, macrophage inflammatory protein 2, and TNF-related apoptosis-inducing ligand mediate the interaction between tumor cells and EPCs, leading to enhanced invasion and angiogenesis [73].

## ECs-immune cells crosstalk in the TME: implications for cancer pathogenesis and therapy

### Understanding the role of ECs in regulating T-cell-mediated anti-tumor responses

T cells are vital for establishing and maintaining adaptive immunity against pathogens, allergens, and tumors [74]. In numerous human malignancies, T cells have been linked with better patient outcomes [75]. However, several drug resistance mechanisms in the TME inhibit or dampen anti-tumor immunity by presenting a range of barriers to T cells [76]. ECs play a crucial role in recruiting and activating T cells as part of the regulation of the immune system [77]. Nevertheless, tumor ECs (TECs) appear to decrease both antigen presentation and immune cell recruitment, resulting in tumor immunosuppression [78]. Combining ICIs with antiangiogenic drugs is increasingly recognized as an effective approach to improve immunotherapy effectiveness and reduce immune-related complications [79]. In murine models, tumor-associated high endothelial venule ECs (TA-HECs) derived from post-capillary venules are recruited into tumors and associated with different types of T cells; enhancement of TA-HECs leads to higher proportions of anti-tumor stem-like CD8^+^ T cells and improves the effectiveness of immune checkpoint blockade (ICB) [80]. Recent research by Sakano et al. indicates that TECs induce tumor-infiltrating T-cell exhaustion through the expression of glycoprotein nonmetastatic melanoma protein B (GPNMB), suggesting that GPNMB could serve as a potential treatment target for liver cancer [81].

TECs are stimulated to express more vascular cell adhesion molecule-1 (VCAM-1) by combined blockade of vascular endothelial growth factor A (VEGFA) and ANG2, which allows anti-tumor T cells to accumulate within various types of tumors in mice [82]. Initially discovered as a cell adhesion molecule, VCAM-1 functions to regulate inflammation-induced vascular adhesion, as well as macrophage and T-cell migration through the endothelium [83]. TECs express programmed cell death 1 ligand 1 (PD-L1), which interacts with programmed cell death 1 (PD-1) present on T cells and inhibits their anti-tumor ability [84, 85]. High expression of PD-L1 on TECs has been shown to reduce CD8^+^ T-cells infiltration, while it increases the aggregate of forkhead box P3 (FOXP3)^+^ Tregs inside tumors; anlotinib can downregulate PD-L1 expression on vascular ECs (VECs) via blocking the AKT pathway [85]. FOXP3^+^ Tregs suppress aberrant immune responses to self-antigens as well as immune responses to tumors, resulting in a poor prognosis when large numbers of Tregs infiltrate tumor tissues [86].

A report by Gkountidi AO et al. suggests that MHC class II-restricted antigen-presenting tumor lymphatic endothelial cells (LECs) suppress anti-tumor immunity by dampening T-cell-mediated responses as well as promoting intratumoral Tregs suppression [87]. Furthermore, previous study has found that PD-L1 is overexpressed by tumor LECs and blood endothelial cells (BECs) in the presence of Interferon-γ (IFN-γ), which inhibits the accumulation of CD8^+^ T cells in TME [88]. IFN-γ is involved in enhancing anti-tumor immunity and protumor immune responses, making it an attractive target for immunotherapeutic interventions [89].

### Targeting TECs to optimize T-cell-mediated anti-tumor responses: emerging approaches

While chimeric antigen receptor (CAR)-T-cell therapy based on gene-editing technology has achieved significant success in treating hematological diseases, its efficacy in treating solid tumors remains limited [90]. Cell surface markers like selectins on ECs and corresponding T-cell receptors are essential for CAR-T cells to reach the tumor site [91]. As cancer progresses, selectins promote several steps that allow tumor cells to interact with blood constituents such as platelets, ECs, and leukocytes [92]. Furthermore, chemokine receptors present on T cells engage with chemokines on ECs, thereby activating endothelial adhesion molecules (EAMs) and triggering ECs activation [93]. Thereafter, intercellular adhesion molecule-1 (ICAM-1) and VCAM-1 expressed on ECs facilitate stable cell adhesion, further enabling chemokine interactions that result in T-cell diapedesis, i.e., the extravasation of T cells into the tissue [93]. However, TECs become anergic when stimulated with pro-angiogenic factors secreted by tumor cells such as VEGFs and fibroblast growth factors (FGFs), thereby becoming unable to be triggered by inflammation and incapable of activating EAMs [93, 94]. Antiangiogenic agents may overcome TECs’ anergy and improve immunotherapy outcomes [94].

TECs not only inhibit the transport and infiltration of anti-tumor T cells, but they also directly affect immune responses by repressing T-cell responses. Through direct contact between cells mediated by the PD-1/PD-L1 pathway, TECs demonstrate an immunosuppressive effect against tumor antigen-specific CD8^+^ T cells. Notably, the deficiency of PD-L1 in TECs compromised their ability to suppress and induce apoptosis in tumor-infiltrating CD8^+^ T cells, ultimately leading to the inhibition of tumor development in an in vivo model [95]. TECs also induce immune suppressive CD4^+^ T cells that influence CD8^+^ T cells through interleukin 10 (IL-10) and transforming growth factor-β (TGF-β) levels, which contribute to tumor immune evasion [95]. Fas ligand (FasL, CD95L) is a key component of programmed cell death [96], and in response to VEGF, IL-10, and prostaglandin E2 (PGE2), TECs are induced to express FasL, which subsequently kills effector CD8^+^ T cells, but not Tregs due to increased cellular-FLICE inhibitory protein (c-FLIP) expression in Tregs [97]. T-cell immunoglobulin and mucin domain 3 (TIM3), originally detected on Th1 cells, contributes to apoptosis in Th1 cells [98]. Extensive studies have elucidated the pivotal role of TIM3 in autoimmune diseases, chronic viral infections, and tumors [98]. In the context of cancer, the interaction between Galectin-9 and PD-1, as well as TIM3, plays a crucial role in regulating T-cell apoptosis, making it a promising target for cancer immunotherapy [99]. The expression of TIM3 in lymphoma-derived ECs contributes to the onset, development, and dissemination of lymphoma by inhibiting CD4^+^ T-cell activation, as well as Th1 polarization [100].

It has been shown that the release of VEGF from oral squamous cell carcinoma (OSCC) cells causes ECs to increase the production of VEGF and PGE_2_, inhibiting T cells [101]. As well, in Lewis lung carcinoma (LLC), tumor secretion of VEGF causes ECs to produce PGE_2_ that suppresses T-cell function [102, 103]. Several cancers express galectin-1 (Gal-1), an immunosuppressive molecule that suppresses immune cell function in TME, contributing to tumor immune evasion [104]. Gal-1 expressed by ECs inhibits T-cell transendothelial migration induced by prostate cancer cells [105]. Targeting Gal-1 may provide several advantages as a potential therapeutic option for cancer; vaccination against Gal-1 promotes cytotoxic T-cell infiltration, which reduces melanoma burden, and also induces VEGF-like signals [106].

CD4^+^ T helper 17 cells (Th17), a relatively new subtype of adaptive immune cells, play a crucial role in defending against extracellular bacteria and fungi invasion [107]. Interleukin-22 (IL-22), a crucial cytokine present in Th17 cells, facilitate the angiogenesis of tumors by acting on ECs [108]. The tumor-penetrating peptide iRGD have shown considerable potential as delivery moieties for improving the penetration of chemotherapeutic agents through angiogenetic vessels into the tumor [109]. iRGD-anti-CD3, a novel bifunctional agent, immobilizes iRGD on T cells by interacting with CD3, causing the formation of vesiculovacuolar organelles (VVOs) in the endothelial cytoplasm, facilitating T-cell migration through the ECs body [110]. Treatment of T cells with iRGD-anti-CD3 significantly inhibited cancer cell development and improved survival in different murine xenograft models, which was further improved by the addition of PD-1 blockade [110].

### Unveiling the potential of NK cell therapy: unraveling the intricacies of NK receptor-TEC interactions

While natural killer (NK) cells have demonstrated remarkable abilities in eliminating cancer cells, their efficacy against solid tumors remains a challenge due to the inhibitory effects of the TME on NK cell activity [111]. Unlike T cells, NK cell activation is governed by the interaction between NK receptors and target cells, which occurs independently of antigen preparation and transmission [112]. A prior study revealed that RAE-1ε, a protein expressed by lymph node endothelial cells (LNECs) and strongly expressed by TECs, activates NKG2D, causing internalization of NKG2D from the NK cell surface and transmitting an NK-intrinsic signal that desensitizes NK cells function, ultimately leading to impaired anti-tumor effects in vivo [113]. However, it was discovered that IL-15-activated NK cells could effectively kill TECs expressing high levels of poliovirus receptor (PVR, CD155) and nectin cell adhesion molecule 2 (Nectin-2), while DNAX accessory molecule-1 (DNAM-1) actively participated in target recognition [114]. It was found that EBV-positive NK lymphoma cell lines produce large amounts of TNF-α which cause elevated levels of ICAM-1 and VCAM-1 in cultured human ECs, and NK cells exhibited enhanced adhesion to cultured ECs that had been treated with TNF-α or IL-1β, and the pretreatment of cytokine-stimulated ECs with anti-VCAM-1 antibodies decreased adhesion [115]. Moreover, cytokines such as TNF-α and IL-1β produced by CD11b^+^ cells in the TME activate ECs to generate CCL2 and CCL7 that recruit NK cells, and induce the expression of ICAM-1 and VCAM-1, allowing ECs to make steady contact with NK cells [116].

CAR-NK cells hold immense promise in the realm of cancer immunotherapy, thanks to their superior safety profile and encouraging outcomes in preclinical and clinical studies [117]. Specifically, an antigen known as CD123 is expressed on ECs, which CAR-T-cell therapy targeting CD123 (CART123) can specifically recognize and attack [118]. However, CART123 can potentially cause injury to ECs, leading to cytokine release syndrome and capillary leakage syndrome, and upregulation of CD123 by IFN-γ and TNF-α can exacerbate these conditions [118]. In contrast, CD123-targeted CAR-NK cells (CAR.CD123-NK cells) offer a safer alternative to CART123, devoid of hematopoietic toxicity or endothelial injury. In an in vivo model, it has been observed that CAR.CD123-NK cells and CART123 cells exhibit distinct toxicity profiles in this particular context [119]. The outcomes of this model revealed that CAR.CD123-NK cells do not exhibit targeting capabilities towards human ECs, thereby preserving the integrity of the vasculature in the murine model. However, the underlying mechanism behind this phenomenon remains undisclosed.

### Interactions between TAMs and ECs in cancer progression: implications for angiogenesis and lymphangiogenesis

Tumor-associated macrophages (TAMs), the most diverse immune cells in the TME, comprise M2 macrophages and a relatively low proportion of M1 macrophages [120]. Whereas M1 macrophages have traditionally been viewed as anti-tumor cells, M2 macrophages participate in several pro-tumorigenic processes in cancer by regulating angiogenesis and lymphangiogenic activity, suppressing the immune system, inducing hypoxia, and promoting tumor cell proliferation and metastasis [121]. In recent years, TAMs have become a promising target for developing new cancer treatments given their close association with malignant tumors [122]. For instance, WNT7b produced by TAMs has been shown to play a crucial role in tumor progression by increasing VEGFA expression in TECs [123]. In addition, TAMs expressing Tie2 are capable of mimicking vascular structure through the expression of EC-related markers and the formation of capillary-like structures in response to VEGF, potentially providing a pathway for vessel maturation with replacement by true ECs [124]. Interaction between TAMs and ECs may enhance endothelial affinity and permeability, thereby facilitating the adhesion and transmigration of circulating tumor cells into tissues and organs [125].

TAMs promote migration of tumor cells via several mechanisms, including the enhancement of endothelial permeability via the release of interleukin-6 (IL-6), C-C motif chemokine ligand 2 (CCL2), and matrix metalloproteinases (MMPs), attaching to VCAM-1 on tumor cells, and suppressing anti-tumor immune cell infiltration [125]. Notably, endothelial permeability refers to the role of ECs as the inner lining of all blood vessels in maintaining organ integrity by regulating tissue perfusion [126]. The angiopoietin-Tie2 signaling axis is also believed to contribute to both regulation and dysregulation of endothelial permeability in the body [126].

Furthermore, gene deletion of Neuropilin-1 (Nrp1) in TAMs reduces their pro-angiogenic and immunosuppressive functions, resulting in a reduction in tumor growth and metastasis [127]. In epithelial ovarian cancer (EOC), TAM infiltration has been found to enhance IL-8 expression in cancer cells, thereby accelerating tumor progression through EC-mediated interactions [128]. TAM-derived exosomes have also been shown to inhibit ECs migration via the miR-146b-5p/TRAF6/NF-kB/MMP2 pathway, while EOC-derived exosomes reverse this process [129]. In addition, M2 macrophage-derived exosomal miR-155-5p and miR-221-5p play critical roles in interactions between TAMs and ECs, thus facilitating the development of pancreatic ductal adenocarcinoma (PDAC) [130]. It was also found that M2 macrophage-derived exosomes suppressed E2F2 expression in ECs, thus promoting angiogenesis [100].

In several tumor types, depletion of TAMs decreases VEGFC production and VEGFR3 signaling in LECs, impairing lymphangiogenesis [131]. Moreover, a subset of TAMs express high levels of podoplanin (PDPN), promoting the attachment of this TAM subset to LECs, thereby promoting the growth of vessels and lymphoinvasion [132]. In the TME of breast cancer, miR-1420-5p, miR-183-5p, and miR-222-3p released from TECs via extracellular vesicles (EVs) lead to the local increase of TAMs and contribute to tumor growth [133]. In addition, Calmodulin2 (CALM2) has been found to exhibit high expression in gastric cancer (GC) cells, modulating the JAK2/STAT3/HIF-1/VEGFA axis, promoting macrophage polarization, and facilitating ECs angiogenesis [134].

### ECs and other immune cells crosstalk: mechanisms driving TME

B lymphocytes, a distinct adaptive immune cell population within the TME, possess intricate and enigmatic functionalities [135]. The B-cell-activating factor of the TNF family (BAFF) is an indispensable factor in hematological, lymphoid carcinomas and immunological domains [136]. Microvascular ECs (MVECs), which constitute the stroma of chronic lymphocytic leukemia (CLL), endow significant abundance. MVECs produce BAFF upon anomalous surface CD40L expression on CLL B cells, suggesting an intimate crosstalk between neoplastic B cells and MVECs [137]. Signal transducer and activator of transcription 3 (STAT3) plays a pivotal role in ECs’ angiogenesis in tumors [138]. Notably, STAT3-dependent stimulation of EC function by B cells significantly boosts tumor angiogenesis [139]. The high-mobility group box 1 (HMGB1) released by cancer cells is known to attract B cells to the tumor in the esophageal squamous cell carcinoma (ESCC), where B cells undergo rapid multiplication and activate pro-angiogenic phenotypes, promoting the growth of both ECs and tumors [140].

Myeloid-derived suppressor cells (MDSCs), notorious for their potent immunosuppressive properties [141], play crucial roles in tumor angiogenesis, drug resistance, and metastasis [142]. Notably, TEC-specific *Shb* deprivation enhances MDSC recruitment and facilitates breast cancer transmission to the lungs in mice [143].

Dendritic cells (DCs), distinguished leukocyte populations capable of initiating and regulating adaptive immune responses, are critical targets of cancer immunotherapy [144, 145]. Deletion of serine/threonine kinase 11 (*Stk11*) in mouse ECs resulted in severe reductions of mature DC numbers and spontaneous tumor formation, indicating the crucial role of DCs in suppressing tumorigenesis [146]. Endothelial-like differentiation (ELD) of DCs represents a poorly studied mechanism contributing to tumor angiogenesis [147]. Notably, within the TME of ESCC, MAPK/ERK1/2 signaling induces immature DCs (iDCs) to undergo ELD, leading to their differentiation into endothelial-like cells instead of mature DCs [148]. This reduces their ability to present antigens, potentially compromising the efficacy of anti-tumor immune responses [149].

The mast cells (MCs) are known to regulate multiple aspects of tumor biology, including cell proliferation and survival, angiogenesis, invasiveness, and metastasis [150]. Upon activation of c-KitR/SCF, MCs release tryptase that acts on PAR2 in both ECs and tumor cells, thereby triggering the proliferation of these cells and promoting tumor invasion and metastasis [151].

**Fig. 2:**
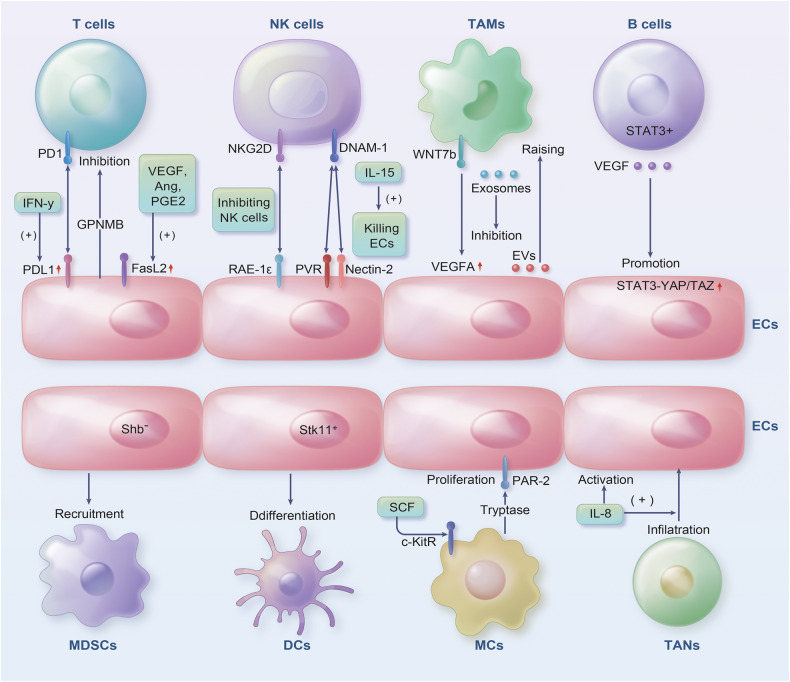
The ECs and immune cells present in the TME engage in intricate interactions. ECs induce tumor-infiltrating T-cell exhaustion through GPNMB. ECs express PD-L1, which binds to PD-1 present on T cells and inhibits their anti-tumor ability. PD-L1 was overexpressed by ECs in the presence of IFN-γ, inhibiting CD8^+^ T-cell accumulation within the TME. In response to VEGF, IL-10 and PGE2, ECs were induced to express FasL, which killed effector CD8^+^ T cells. NKG2D in NK cells is activated by RAE-1ε expressed from ECs, inhibiting the anti-tumor effects of NK cells. When DNAM-1 recognizes PVR and Nectin-2 on ECs, IL-15 activates NK cells to kill ECs. WNT7b produced by TAMs increases VEGFA expression in ECs. MiR-1420-5p, miR-183-5p and miR-222-3p released from TECs via EVs lead to local TAM increases. TAM-derived exosomes inhibit ECs migration via the miR-146b-5p/TRAF6/NF-kB/MMP2 pathway. VEGF produced in B cells activates STAT3 and promotes YAP/TAZ interaction, leading to ECs progression. The lack of *Shb* in ECs led to MDSCs recruitment. ECs with *Stk11* deletion reduce mature DC numbers and spontaneous tumor formation. As a result of activation of c-KitR/SCF, MCs release tryptase, acting on PAR2 in ECs, triggering ECs proliferation. Due to IL-8 signaling, ECs are promoted, TANs infiltrating tumor sites migrate more efficiently.

Tumor-associated neutrophils (TANs) are crucial in cancer development, as well as resistance to or response to therapy [152]. A distinction exists between N1 and N2 TANs based on their activation status, cytokine status, and effects on cancer cell development. N1 TANs exhibit anti-tumor properties, while N2 TANs promote immunosuppression, cancer development, angiogenesis, and metastasis [153]. The diverse effectors and downstream targets involved in IL-8 signaling promote ECs’ angiogenic responses, increase the proliferation and survival of both ECs and cancer cell, and enhance the migration efficiency of cancer cells, ECs, and TANs infiltrating tumor sites [154] (Fig. 2).

## ECs-related immune checkpoints in the TME: implications and mechanisms

ICB is an immunotherapy approach that inhibits tumor-mediated suppression of anticancer immune responses, as opposed to strategies that directly damage tumor cells [155]. Two prominent approaches to checkpoint-blocking include blocking cytotoxic T-lymphocyte-associated protein 4 (CTLA-4) and targeting the interaction between PD-1 and PD-L1 [156]. Both immune and non-immune cells, including ECs of the lungs, liver, and small intestine, express PD-1 and PD-L1 [157]. Previous evidence indicated that PD-L1 expression in ECs reduces the activation and cytolysis of CD8^+^ T cells [158]. Moreover, it may augment both Tregs activation and cytokine production [159]. The presence of TME appears to promote the expression of PD-L1 in ECs, with VEGFA and hypoxia playing a pivotal role in this process [85]. Previous research suggests that the regulation of PD-L1 in ECs may be crucial for modulating immune responses [160]. Anlotinib in particular, suppresses PD-L1 expression in TECs and retards tumor cell proliferation [85].

The negative regulation of T-cell responses is a crucial function of CTLA-4 [161]. In the context of melanoma treatment, the inhibitory signals between antigen-presenting cells and T cells controlled by the CTLA-4 molecule can be blocked by anti-CTLA-4 [162]. Notably, anti-CTLA-4 treatment-induced normalization of tumor vessels was found to be accompanied by an increase in eosinophil infiltration into breast tumors, and a positive correlation emerged between the accumulation of eosinophils and the degree of responsiveness exhibited by breast tumors towards anti-CTLA-4 treatment [163]. Among the first targeted therapies and angiogenesis inhibitors, bevacizumab is a monoclonal antibody that targets VEGFA [164]. In contrast, ipilimumab, a human monoclonal antibody, blocks the immune checkpoint CTLA-4. Interestingly, the combination of ipilimumab and bevacizumab resulted in the activation of TECs [165]. Furthermore, in vitro studies revealed that the combination of ipilimumab and bevacizumab promoted the expression of E-selectin, ICAM-1, and VCAM-1 on TECs, as well as the adhesion of activated T cells to TECs by increasing the levels of IL-1α and TNF-α [166].

As an immune checkpoint on T cells, TIM3 plays a pivotal role in tumor immune response. Co-blockade of TIM3 and PD-1 has been shown to induce tumor regression in preclinical models and enhance anticancer T-cell responses in advanced cancer patients [167]. In various cancers, TIM3 and Galectin-9 (Gal9) interact to suppress both innate and adaptive immunity [168]. However, despite the established role of TIM3 as a receptor for galectin-9, a previous study have unveiled an intriguing phenomenon. In a melanoma model, it has been demonstrated that the TIM3 ECs promote tumor cell proliferation, survival and migration by activating NF-κB in tumor cells in a galectin-9-independent manner [169]. The expression of TIM3 in lymphoma-derived ECs facilitates lymphoma development and spread by interfering with circulating T cells and inhibiting CD4^+^ T-cells activation [100]. Clinically, the presence of TIM3 in the endothelium of B-cell lymphomas has been associated with the disease spread and poor patient outcome [100]. It has also been reported that overexpression of TIM3 in breast cancer cell lines leads to accelerated tube formation of ECs due to upregulated VEGF [170]. Thus, TIM3 is likely to play a crucial role in regulating angiogenesis and endothelial-related diseases in the human body [171].

The B7-H3 protein (also called CD276), an immune checkpoint protein, belongs to the B7 family and is a critical regulator of the adaptive immune response [172]. It is also considered an emerging key player in the development of cancer. B7-H3 is highly expressed in differentiated malignant cells and cancer-initiating cells, making it a valuable target for antibody-based immunotherapy [173]. In addition, B7-H3 has been identified as the surface marker for TECs, which can distinguish between physiological and pathological angiogenesis [174]. Notably, TECs expression of B7-H3 has been found to predict prognosis in ovarian carcinomas [175] and renal cell carcinomas (RCC) [176]. By activating the NF-κB pathway, B7-H3-overexpressing colorectal cancer cells increase the expression of VEGFA, which promotes ECs angiogenesis [177]. Conversely, a study conducted in the breast cancer cell line MCF-7 found that silencing B7-H3 enhanced the production of VEGF [178]. Furthermore, a correlation has been observed between the expression of B7-H3 in Merkel cell carcinoma-associated ECs and both local aggressive primary tumor characteristics and an increase in vascular density [179].

The CD40 receptor and its ligand CD40L comprise a crucial molecular pair of the stimulatory immune checkpoint [180]. CD40 and CD40L interact to mediate anti-tumor immune responses by increasing immunogenic cell death (ICD) of tumor cells, activating antigen-presenting cells, producing proinflammatory factors and stimulating CD4^+^ and CD8^+^ T cells [181]. CD40 is expressed by various cells, including B cells, monocytes, DCs, fibroblasts, tumor cells and ECs [182, 183]. Interestingly, CD40/CD40L binding induces leukocyte adhesion to ECs through the E-selectin pathway while inhibiting ECs migration through blocking the Akt/eNOS pathway [184]. Breast cancer development has been linked to CD40 activation on ECs, and activated platelets, which can interact with and activate ECs, may act as a source of CD40L [185]. In a metastatic breast cancer model, CD40L-expressing EPCs exhibit anti-tumor properties by stimulating the secretion of both TNF-α and IFN-γ [186]. While CD40-stimulating immunotherapy can enhance anti-tumor responses by activating DCs and increasing T-cells priming, TECs increase Indoleamine 2, 3-dioxygenase 1 (IDO-1), which has an immunosuppressive feedback mechanism that inhibits the response to such immunotherapy [187]. When combination with sunitinib, anti-CD40 mAb treatment increased the expression of ICAM-1 and VCAM-1 on TECs, resulting in enhanced intratumoral infiltration of CD8^+^ cytotoxic T cells [188].

CD137, also known as 4-1BB, is a member of the TNF receptor superfamily and functions as a costimulatory immune receptor [189]. This crucial receptor facilitates the activation of CD8^+^ T cells and additionally triggers NK cells and DCs, thereby eliciting further activation of cytotoxic T cells [190]. CD137 is expressed by a variety of cells such as T cells, B cells, NK cells, DCs, eosinophils, and MCs [191]. When CD137 is present on the surface of TECs, treatment with CD137 monoclonal antibodies (mAbs) leads to upregulated expression of ICAM-1, VCAM-1, and E-selectin, promoting recruitment of CD8^+^ T cells into the malignant tissue and engendering an immune response against the cancerous cells [192].

CD278 (also known as ICOS or inducible T-cell costimulatory factor) represents an essential immune checkpoint that becomes activated and expressed on T cells [193]. The expression of ICOS ligand (ICOSL) can be found on many different types of antigen-presenting cells, including macrophages, DCs, and B cells, non-hematopoietic cells, and ECs [194]. The binding of ICOS and ICOSL produces many activities among the diverse subpopulations of T cells, including activation, effector function, and suppression [195]. Previous studies have demonstrated that the interaction between Tregs and ECs via ICOS/ICOSL-mediated signaling pathways increases the sensitivity of B-lymphoma cells towards ABT-199 [196]. In addition, research supports that ICOSL acts as an uncharacterized receptor for osteopontin, and their interaction promotes ECs and tumor cell migration [197]. In vitro experiments have demonstrated that ICOSL, stimulated by a soluble recombinant form of ICOS (ICOS-Fc), effectively prevents the adhesion and migration of DCs, ECs, and tumor cells [198].

Indoleamine 2,3-Dioxygenase (IDO), an enzyme produced by various immune cells, stromal cells, and tumor cells, has been implicated in the suppression of effector T cells and the promotion of Tregs proliferation [199]. Notably, the expression of IDO-1 in TECs is linked to the efficacy of immunotherapy in metastatic RCC patients [200]. Within RCC ECs, IDO may limit tryptophan flowing into tumors or generate tumor-toxic metabolites, ultimately restricting tumor proliferation and improving patient prognosis [201]. Conversely, high levels of IDO have been associated with poorer outcomes in breast cancer and significant ECs proliferation in vitro [202]. Research has also demonstrated that miR-142-5p transferred into LECs through tumor cell-secreted exosomes induces lymphatic IDO expression and exhausts CD8^+^ T cells [203].

The phagocyte NADPH oxidase isoform 2 (NOX2) is a critical enzyme involved in antigen presentation and immune regulation [204]. Recent studies suggest that reactive oxygen species (ROS) produced by NOX2^+^ myeloid cells in the TME play multiple roles in cancer cell proliferation and metastasis [205]. By generating ROS, NOX2 degrades adjacent cytotoxic lymphocytes such as NK cells and T cells, resulting in impaired function and viability [206]. The expression of NOX1 and NOX2 in melanoma-conditioned media promotes the EndMT progression of ECs [207]. NOX2 expression in early and late endosomes of ECs produces ROS that regulate cell proliferation, promoting angiogenesis in prostate tumors [208]. In vivo, tumor growth derived from ECs has been shown to be inhibited by NOX2 and NOX4 inhibitors [209].

Since TNF receptor 2 (TNFR2) plays a crucial role in TME, it has emerged as a highly promising immune checkpoint for targeted therapy [210]. Indeed, many human tumors are known to exhibit high levels of TNFR2 expression [211]. Interestingly, TNFR2 protein is also highly expressed by Tregs, and its activation and proliferation greatly contribute to the survival and growth of cancer cells [212]. Through the TNFR2/Akt and ERK signaling pathways, colorectal cancer-derived progranulin (PGRN) activates EC proliferation and angiogenesis [213]. The EC colony-forming units (EC-CFUs) from patients with breast cancer exhibit reduced expression of genes related to TNFR2 signaling, which results in resistance to apoptosis mediated by TNF-α [214]. Evidence has also emerged that TNF-α may enhance myeloma cell migration across ECs via its interaction with TNFR2 and its effect on autocrine activation of MCP-1 [215]. These findings highlight the considerable promise of therapies targeting TNFR2 in cancer treatment.

**Fig. 3:**
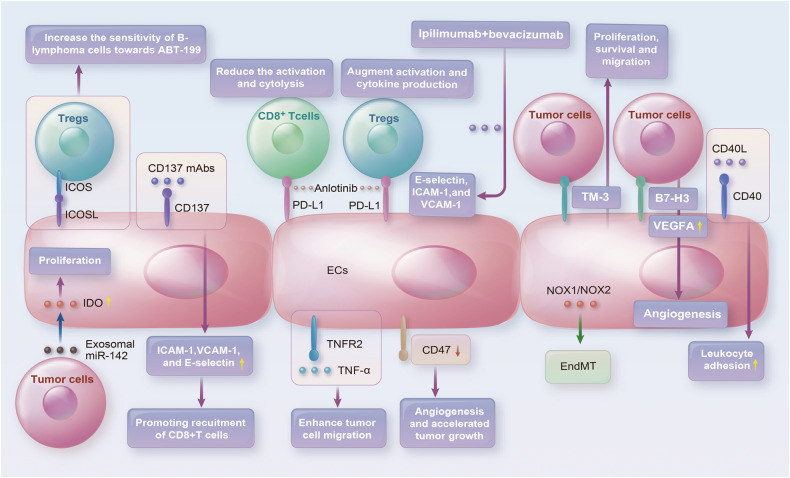
Immune checkpoints associated with ECs interact with the TME to affect the tumor in a variety of ways. In ECs, PD-L1 reduces CD8^+^ T-cell activation and cytolysis and increases Treg activation and cytokine production. Anlotinib inhibits PD-L1 expression in ECs. Ipilimumab blocks CTLA-4 and activates ECs. Overexpression of Tim3 in tumor cells leads to ECs tube formation acceleration. B7-H3-overexpressing tumor cells promote ECs angiogenesis. CD40/CD40L binding induces leukocyte adhesion to ECs. CD137 is present on ECs surfaces, and treatment with CD137 mAbs leads to increased recruitment of CD8^+^ T cells. ICOS/ICOSL-mediated interactions between Tregs and ECs increase the drug sensitivity of tumor cells. MiR-142-5p is transferred into ECs through tumor cell-secreted exosomes to exhaust CD8^+^ T cells through an increase in IDO expression. NOX2 promotes EndMT progression of ECs. TNF-α, through TNFR2 enhances tumor cell migration across ECs. A function-blocking CD47 antibody B6H12 modulates multiple EVs-mediated signals between tumor cells and ECs that are critical for tumor cell proliferation and metastasis.

CD47 has emerged as an important macrophage immune checkpoint that is overexpressed by many cancer cells, enabling tumors to evade macrophage phagocytosis [216]. Studies suggest that inhibiting the interaction between signal-regulatory protein α (SIRPα) and CD47 may enhance the ability of macrophages to eliminate tumor cells [217]. Interestingly, the downregulation of CD47 in TECs has been associated with increased angiogenesis, suppressed tumor necrosis formation, and accelerated tumor growth [218] (Fig. 3). Moreover, a function-blocking CD47 antibody B6H12 is capable of modulating multiple EVs-mediated signals between breast tumor cells and ECs that promote the proliferation and metastasis of tumor cells [219]. Additionally, ECs exhibit radioresistance in vitro and protect soft tissue, bone marrow, and tumor-associated leukocytes in irradiated mice by blocking CD47 signaling [220]. These findings underscore the potential of CD47-based therapies in cancer treatment and support further investigations into their clinical translation.

## Preventing angiogenesis: targeting ECs for enhanced therapeutic efficacy

### Advancements and prospects in therapeutics targeting ECs

The process of angiogenesis, which involves the formation of new blood vessels, plays a pivotal role in tumor progression and metastasis. Several approaches have been explored to specifically target ECs in cancer therapy. One approach involves using antiangiogenic agents that directly inhibit the formation of new blood vessels. Most of these agents disrupt the VEGF signaling pathways involved in ECs proliferation and migration, effectively impeding tumor growth [221]. Another strategy is to utilize targeted therapies that specifically bind to receptors expressed on ECs. By selectively delivering cytotoxic agents or immune modulators to the tumor vasculature, these therapies aim to induce vessel normalization, enhance immune responses, and improve drug delivery to the tumor site [222]. Furthermore, emerging research has explored the use of nanotechnology-based approaches to target ECs. The nanoformulated STING activator ZnCDA, based on the NCP platform, selectively activates ECs, leading to disruption of tumor vasculature and improved targeted drug delivery [223]. Nanoparticles offer the potential for increased therapeutic efficacy while minimizing off-target effects.

Clinical trials investigating the targeting of ECs in cancer therapy have shown promising results in various cancer types, such as breast [224], lung [225], and colorectal cancer [226]. These studies have demonstrated improved patient outcomes. Despite these advancements, challenges remain in effectively targeting ECs for cancer therapy. Identifying specific markers and pathways unique to ECs, optimizing drug delivery systems, and overcoming resistance mechanisms are areas of ongoing research.

### Synergistic effect of ICB and anti-angiogenesis in cancer treatment

VEGF and VEGFR represent pivotal regulators of tumor angiogenesis and proliferation. Agents precisely targeting these molecules have been extensively investigated for their potential in addressing diverse cancer types. The addition of bevacizumab to combination chemotherapy for recurrent, persistent, or metastatic cervical cancer resulted in improved overall survival [227]. In recent years, researchers have embarked upon the exploration of synergistic combinations, specifically integrating VEGF and VEGFR-targeted therapeutics with ICIs [228]. This novel therapeutic approach aims to magnify treatment outcomes by concurrently inhibiting tumor angiogenesis and potentiating the anti-tumor functionality of the immune system. Notably, clinical trials have showcased substantial improvements in overall survival and amelioration of disease burden in individuals afflicted by non-small cell lung cancer [229] and renal cell carcinoma [230]. As depicted in Table 1, a multitude of ongoing clinical trials are focused on investigating the synergistic impact achieved through the combination of anti-angiogenesis drugs with immunotherapy.

**Table. 1:** The integration of antiangiogenic agents with immunotherapeutic modalities in ongoing clinical trials.

NCT number	Study phase	Study status	Cancer type	Interventions
NCT04211168	II	RECRUITING	Biliary tract cancer	Toripalimab + Lenvatinib
NCT05733611	II	RECRUITING	Colorectal cancer	RP2/RP3 + Atezolizumab + Bevacizumab
NCT04721132	II	RECRUITING	Hepatocellular carcinoma	Atezolizumab + Bevacizumab
NCT04444167	I/II	RECRUITING	Hepatocellular carcinoma	AK104 (Anti-PD-1/CTLA-4 bispecific antibody) + Lenvatinib
NCT04443309	I/II	RECRUITING	Hepatocellular carcinoma	Camrelizumab + Lenvatinib
NCT04356729	II	RECRUITING	Melanoma	Atezolizumab + Bevacizumab
NCT05756972	II/III	RECRUITING	Non-small cell lung cancer	PM8002 (Anti-PD-L1/VEGF bispecific antibody)/Placebo + Carboplatin + Pemetrexed
NCT04749394	II	RECRUITING	Non-small cell lung cancer	Camrelizumab (PD-1 monoclonal antibody) + Apatinib (VEGFR2 antibody)
NCT04878107	II	RECRUITING	Non-small cell lung cancer	Camrelizumab + Apatinib + Stereotactic body radiation therapy + Low dose radiation therapy
NCT04919629	II	RECRUITING	Ovarian, fallopian tube, or primary peritoneal cancer	Bevacizumab + Pegcetacoplan + Pembrolizumab
NCT04981509	II	RECRUITING	Renal cell carcinoma	Atezolizumab + Bevacizumab + Erlotinib
NCT05116007	I	RECRUITING	Small cell lung cancer	AK112 (Anti-PD-1/VEGF bispecific antibody) + Etoposide + Carboplatin

Data were acquired from the U.S. National Library of Medicine (http://clinicaltrials.gov, accessed on August 10, 2023).

However, a multitude of unresolved queries persist. Paramount among these are the delineation of the optimal combinational therapeutic regimens, the identification of suitable patient cohorts, and the judicious management of potential adverse events stemming from these combined interventions. Conclusively, the integration of VEGF and VEGFR-targeted therapeutics with ICIs represents a captivating treatment paradigm that holds immense promise in select oncological contexts.

## Conclusions

ECs are critical in tumor development and metastasis, and their relationship with immune cells is essential for the immune response to tumors. It is essential to understand the dynamics between immune cells and ECs to improve the efficacy of immunotherapy. The specific targeting of ECs offers a potent means to effectively impede angiogenesis and suppress the growth of tumors. By selecting appropriate targets such as VEGF, highly specific therapeutic effects can be achieved.

Immune cells influence ECs and regulate angiogenesis in various ways, emphasizing the crucial role of the interplay between these cells in tumor development. Recent insights into the role of immune checkpoints and ECs in immune function have resulted in highly promising anti-tumor therapies targeting these pathways for cancer treatment. Various therapeutic strategies aimed at modulating ECs and immune cells were meticulously scrutinized, encompassing the utilization of monoclonal antibodies, cell-based vaccines, and ICIs.

ECs downregulate antigen presentation and recruitment of immune cells, contributing to immunosuppression. Thus, targeting ECs may assist in improving the immune effect of immune cells in TME. It is crucial to regulate the markers, receptors, and signaling pathways associated with ECs to enhance the effectiveness of immunotherapies like CAR-T-cell therapy and ICIs.

Furthermore, it is imperative to acknowledge the challenges and limitations that beset these therapeutic interventions, including the emergence of treatment resistance and the intricate mechanisms of immune evasion. The development of effective immune targets and improvement of ICIs can be achieved through further study of the effects of these pathways on the immune microenvironment. Understanding the interplay between ECs and immune cells can provide important insights into the mechanisms of tumor progression and help design effective anti-tumor therapies. Future studies may focus on developing novel methods for harnessing the power of the immune system to eliminate tumors.

In light of the recent advances and insights presented in this review, it is unequivocal that the intricate partnership between endothelial cells, immune cells, and immune checkpoints plays a critical role in shaping the TME. This partnership is orchestrated by complex molecular and cellular mechanisms that exert a profound impact on tumor growth, metastasis, and response to treatment. We have gained new insights into the mechanisms of immune evasion employed by tumors, as well as the molecular and cellular components that drive angiogenesis and immune modulation in the TME. In conclusion, this review unveils the stunning complexity and beauty of the TME, revealing a new paradigm for understanding and treating cancer.

## Data Availability

All data in this review are available.
